# The distribution and function of human memory T cell subsets in lung cancer

**DOI:** 10.1007/s12026-016-8882-y

**Published:** 2017-01-19

**Authors:** Si Yuan Sheng, Yong Gu, Chuan Gang Lu, Jian Yong Zou, Hai Hong, RongFu Wang

**Affiliations:** 1 Key Laboratory of Tropical Disease Control of Sun Yat-Sen University, Ministry of Education, The Institute of Immunology of Zhong Shan Medical School, Sun Yat-Sen University, No. 74 Zhong Shan Two Road, Guang Zhou, Guang Dong 510000 China; 2 The First Affiliated Hospital of Sun Yat-Sen University, No. 58 Zhong Shan Two Road, Guang Zhou, Guang Dong 510000 China; 3 Houston Methodist Research Institute, Houston, TX USA

**Keywords:** Human memory T cell, Lung cancer

## Abstract

The distribution and function of T lymphocytes in human lung cancer remain limited. In this study, we investigated the properties of human T cell subsets in the blood of non-small cell lung cancer (NSCLC) patients. We found a relatively normal level of CD4+ subsets in the blood of NSCLC patients, but CD8+ effector T cells increased and CD8+ effector memory cells declined compared to the healthy donors. To further analyze their properties, we stimulated the peripheral blood mononuclear cells (PBMCs) of NSCLC patients by mitogens to examine cytokine production. Our data suggest that both CD4+ and CD8+ naïve cells in NSCLC patients significantly reduced IFN-γ and TNF-α production. Additionally, fewer CD8+ effector cells produced IFN-γ and TNF-α in NSCLC patients than in healthy subjects. Moreover, similar results were observed for CD4+ or CD8+ memory cells in NSCLC patients for the production of IFN-γ, TNF-α, and IL-17. Therefore, our results strongly suggest that the function of CD4+ and CD8+ T lymphocytes in NSCLC patients is compromised or dysregulated. The development of vaccines and antitumor immunotherapy may be essential for the treatment of lung cancer patients.

**Electronic supplementary material:**

The online version of this article (doi:10.1007/s12026-016-8882-y) contains supplementary material, which is available to authorized users.

## Introduction

Immunological memory is critical for long-term immunity and protection from infection. After naïve T cells are activated by the antigen, naïve T cells differentiate into effector T cells, depending on the anatomical position and phenotypic characteristics; effector T cells display different functions [1]. However, only a small fraction of effector T cells becomes long-lived memory T cell to provide lifelong protection against the previously encountered pathogens [2, 3]. With respect to the tissue homing-related molecular expression, memory T cells can be divided into two categories, central memory T cells (Tcm) and effector memory T cells (Tem) [4]. Recent data revealed that adoptively transferred different subsets of memory T cells have different antitumor activity in mouse models [5]. The distribution and function of human memory T cells have been identified in healthy subjects [6], but the physiological distribution and function of human T cell subsets in lung cancer are still limited. Clearly, the understanding of the compartmentalization of memory T cell subsets will provide valuable basis for designing tumor immunotherapy.

Current studies focus on the frequency of the tumor-infiltrating lymphocytes (TILs) to predict the prognosis of cancer patients [7]. The high frequency of CD4+ T cells in TILs and malignant pleural effusions (MPEs) correlates with a favorable prognosis in lung cancer patients [8, 9]; however, other studies indicate that the high number of CD8+ T cells, not CD4+ T cells, in TILs has a good clinical outcome [10–12]. The distinct distribution of CD4+ and CD8+ T cell in TILs results in different clinical outcomes in lung cancer patients. The presence of high density of CD3+CD8+CD45RO+ immune cells within tumor region is correlated with favorable clinical outcome in epithelial ovarian cancer [13], while the number of effector CD8+ T cells in TILs decreased in lung cancer [14]. In our study, we assessed the expression of memory T cell subsets in non-small cell lung cancer patients, and herein, we show the distinct compartmentalization of naïve T cells (Tn), Tcm, Tem, and effector T cell (Teff) subsets in non-small cell lung cancer (NSCLC). Our results provide further information regarding the distribution and function of CD4+ and CD8+ memory T cell subsets in human NSCLC patients. These results will lead to a better understanding of the biology of lung cancer.

## Material and methods

### Study participants

Eight NSCLC patients from the First Affiliated Hospital of Sun Yat-Sen University of Guangzhou, China, were enrolled in this study. The eight patients including three female and five male have the age range from 41 to 78 years. The final diagnosis of lung cancer was based on pathological evidence (detected by histological staining), one case of stage IV, three cases of stage III, two cases of stage II, and two cases of stage I cancer (Table 2). Patients whose serology tested positive for HIV, HBV, and HCV were excluded from the study. None of the patients received cancer-related chemotherapy during the period of collecting samples. The blood and lymph nodes were collected from the same NSCLC patients. Eight healthy donors were recruited for collecting blood (the age range from 18 to 40 years); healthy lymph nodes were taken from non-lung cancer patients (the age range from 29 to 70 years). The sex of the healthy donor control group and lung cancer group samples was matched.

### Isolation of peripheral blood mononuclear cells and lymphocytes

The lymph nodes were maintained in cold Hanks’ buffer and brought to the laboratory within 2–4 h after surgery. The lymph nodes were cut into small pieces and mashed with cold PBS, after which any residual tissue fragments were removed using a strainer (70 μm) (BD Falcon, 352340). The suspension was centrifuged at 524*×g* for 10 min at RT. The pellets were washed with PBS and then resuspended in complete RPMI 1640 medium (Invitrogen, Grand Island, NY, USA, cat. 11875093) supplemented with 10% heat-inactivated fetal bovine serum (FBS; Invitrogen, Grand Island, NY, USA), 100 U/mL penicillin (cat. 15071163), 100 mg/mL streptomycin (15071163), 2 mM L-glutamine (cat. 25030081), and 50 mM 2-mercaptoethanol (cat. 21985023; Invitrogen, Grand Island, NY, USA). The peripheral blood mononuclear cells (PBMCs) were isolated from sodium heparin-treated blood obtained from healthy donors or the lung cancer patients by Ficoll-Hypaque (Tian Jin Hao Yang Biological Manufacture Co., Ltd., China, cat. LTS1077) gradient centrifugation. The erythrocytes were lysed using an ammonium chloride solution.

### Flow cytometry analysis

#### Phenotypic characterization

The pooled PBMCs and lymph node cells from the healthy donors and the lung cancer patients were stained for flow cytometry. The following panel of mouse anti-human mAbs, all purchased from BD Biosciences (San Jose, CA, USA) or eBioscience (San Diego, CA, USA), was used: anti-human CD3-APC.cy7 (BD, 557832, SK7), anti-human CD4-Percp.cy5.5 (BD, 560650, RPA-T4), anti-human CD45RA-FITC (eBioscience, 11-0458-42, HI100), and anti-human CCR7-PE.cy7 (BD, 557648, 150503). The cell data were acquired using a 10-laser Gallios (Beckman Coulter Inc., Brea, CA, USA) analytical flow cytometer. Unstained and single fluorochrome-stained cells were used as controls to provide accurate compensation and data analysis. The results were analyzed with Kaluza software.

#### Intracellular staining

The PBMCs and lymph node cells were incubated in 96-well bottom plates at 2 × 10^6^ cells per well in RP10 media (RPMI, 10% heat-inactivated FBS) alone or with phorbol 12-myristate 13-acetate (PMA) (20 ng/mL) plus ionomycin (1 μg/mL) for 4 to 6 h at 37 °C in the presence of BFA (10 μg/mL). The cells were harvested, washed with PBS, stained for the surface phenotypic markers, and fixed at RT with 2% PFA. The cells were then permeabilized (0.01% saponin), and the intracellular cytokines were stained using anti-human IFN-γ-V450 (BD, 560371, B27), anti-human IL-17A-PE (BD, 560486, N49-653), and anti-human TNF-α-APC (eBioscience, 17-7349-82, MAB11). All samples were analyzed using a Beckman Gallios instrument. The data were analyzed using the Kaluza software (Beckman Coulter Inc., Brea, CA, USA). PMA (cat. 16561-29-8), ionomycin (cat. 10634), brefeldin A (BFA) (cat. B7651), bovine serum albumin, and NaN_3_ were all purchased from Sigma-Aldrich (St. Louis, MO, USA).

### Statistical analysis

GraphPad Prism software version 5 was used for the statistical analysis. The Mann–Whitney test (two-tailed) and non-paired Student’s *t* test were performed to identify significant differences. A value of *p* ≤ 0.05 was considered statistically significant.

## Results

### The distribution of CD4+ and CD8+ T cell subsets in human lung cancer

The eight lung cancer patients recruited to this study had been diagnosed with NSCLC and were HIV and HBV negative and free of other cancers. We obtained blood from healthy donors (*n = 8*) and collected blood and lymph nodes (LNs) from lung cancer patients. Healthy lymph nodes were taken from non-lung cancer patients (*n* = 6).

To assess the distribution of the CD4+ and CD8+ T cell subsets in human lung cancer, we analyzed the Tn, Tcm, Tem, and Teff of the PBMCs from the healthy donors and NSCLC patients by flow cytometry according to established surface markers (Tables 1 and 2) [1, 2].

**Table 1:** Marker of human primary lymphocyte subsets for flow cytometry

Subsets	Donors (*n*)	Surface marker
CD4+ naive	6–8	CD4+CCR7+CD45RA+CD45RO
CD4+ Tcm	6–8	CD4+CCR7+CD45RA−CD45RO+
CD4+ Tem	6–8	CD4+CCR7−CD45RA−CD45RO+
CD4+ Teff	6–8	CD4+CCR7−CD45RA+CD45RO
CD8+ naive	6–8	CD8+CCR7+CD45RA+CD45RO
CD8+ Tcm	6–8	CD8+CCR7+CD45RA−CD45RO+
CD8+ Tem	6–8	CD8+CCR7−CD45RA−CD45RO+
CD8+ Teff	6–8	CD8+CCR7−CD45RA+CD45RO−

Eight human lymphocyte subsets from normal peripheral blood lymphocytes (*n* = 8), lung cancer peripheral blood lymphocytes (*n* = 8), normal lymph nodes (*n* = 6), and lung cancer lymph nodes (*n* = 8) gated by various surface marker combinations

**Table 2:** Non-small cell lung cancer patient samples

Feature	Cases
Age	
<60	4
>60	4
Gender	
Male	5
Female	3
Histology	
Adenocarcinoma	4
Squamous carcinoma	4
Stage	
I–II	4
III–IV	4

Eight lung cancer patients which comprise five males and three females from 41 to 78 years of age were recruited in this study; all cases were newly diagnosed and had not received anticancer therapy. The study was approved by the Sun Yet-San University ethics committee. Four cases were adenocarcinoma of lung cancer and four cases squamous carcinoma of lung cancer, two cases of stage I, two cases of stage II, three cases of stage III, and one case of stage IV cancer

**Fig. 1:**
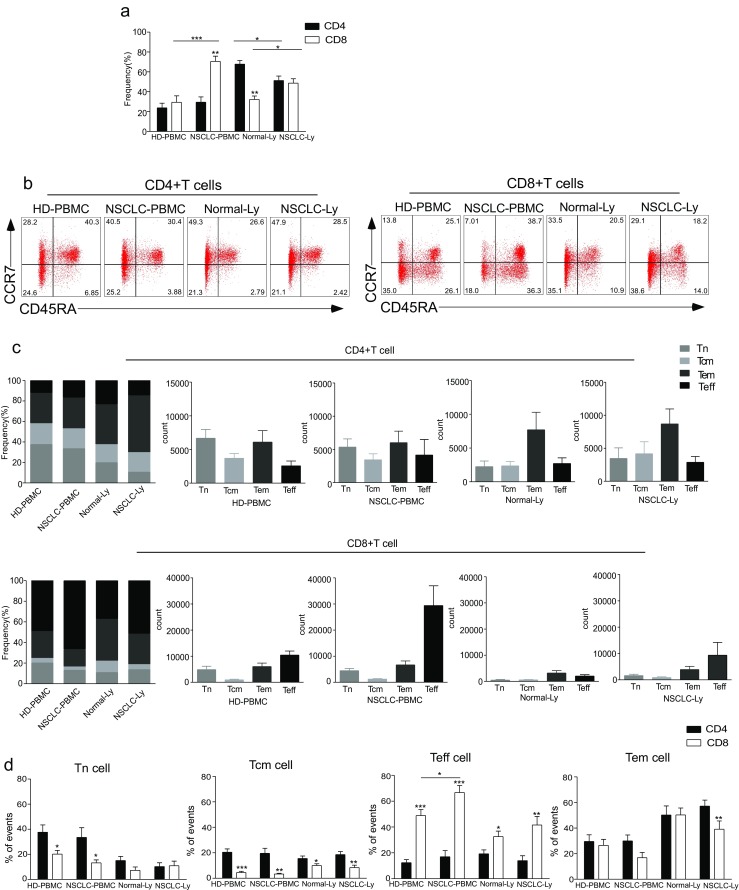
The distribution of CD4+ and CD8+ T cells subsets in human lung cancer. PBMCs were isolated from the blood of lung cancer patients and healthy donors and analyzed by flow cytometry. **a** The frequency of the CD3+CD4+ T cells and CD3+CD8+ T cells in the HD-PBMC, PBMCs from healthy donors; NSCLC-PBMC, PBMCs from non-small lung cancer patients, Normal-Ly, from healthy lymph node, NSCLC-Ly, tumor infiltrated lymph node from non-small lung cancer patients. **b** Representative flow cytometric analyses of CD45RA and CCR7 expression in CD3+CD4+ T cells and CD3+CD8+ T cells, indicating naïve T cells (CD45RA+/CD45RO-CCR7+, *top right quadrant*), terminal effector T cells (CD45RA+/CD45RO-CCR7-, *bottom right quadrant*), central memory T cells (Tcm, CD45RO+/CD45RA-CCR7+, *top left quadrant*), and effector memory T cells (Tem, CD45RO+/CD45RA-CCR7-, *bottom left quadrant*), gated on the forward and side scatter of the lymphocyte populations. **c** The frequency and absolute number of the CD4+ (*top*) and CD8+ (*bottom*), Tn (*middle gray*), Teff (*black*), Tcm (*grey*), and Tem (*dark grey*) cell subsets in the blood from the non small cell lung cancer patients and healthy donors. **d** The events of Tn, Teff, Tcm and Tem cell subsets of CD4+ and CD8+ cells in the blood from non small cell lung cancer patients and healthy donors, expressed as the mean ± SEM. *p < 0.05; **p < 0.005; ***p < 0.001; Mann–Whitney test (two-tailed) and non-paired Student’s t-test

We gated the CD3+CD4+ T cells and CD3+CD8+ T cells in the healthy donors and NSCLC patients. We found that the frequency of CD8+ T cells increased in blood (*p* = 0.0002) and lymph node (*p* = 0.022) from NSCLC patients compared to the normal group, while the frequency of CD4+ T cells declined in NSCLC-Ly group (*p* = 0.022). The CD8+ T cells outnumbered the CD4+ T cells in NSCLC-PBMC (*p* = 0.006); in Normal-Ly, the CD4+ T cells outnumbered the CD8+ T cells (*p* = 0.004) (Fig. 1a). In humans, the CD45RO isoform is the marker that distinguishes between naïve and memory T cells. Naïve cells are CD45RA+ and CD45RO−, while memory T cells are CD45RA− and CD45RO+. We used the LN homing receptor CCR7 to define the subsets of CD45RA+ and CD45RO+ T cells (Fig. 1b). The analysis of the CD4+ T cell subsets indicated that the peripheral blood and lymph node contained similar frequencies and absolute count of the Tn, Tcm, Tem, and Teff cell populations both in the healthy donors and NSCLC patients (Fig. 1c). The CD8+ Teff cells predominated in the peripheral blood, representing 50–70% of the total CD8+ T cells. In the NSCLC patients, the proportion and absolute count of the CD8+ Teff cells were higher in NSCLC-PBMC (70%) than in HD-PBMCs (50%) (Fig. 1c). In contrast, the frequency of CD8+ Tem cells was lower in the blood of the NSCLC patients than in the healthy donors (Fig. 1c). In the NSCLC patients, CD4+ Tem and CD8+ Teff cells predominated in the lymph node (NSCLC-Ly) (Fig. 1c). The subtypes of the CD4+ and CD8+ T cell differed in both the health donor and NSCLC patient. Specifically, the frequencies of the CD4+ Tn (*p* = 0.031) and Tcm cells (*p* = 0.0032) were greater than those of the CD8+ T cells in the blood of the NSCLC patients; the proportion of the CD4+ Tcm was greater than that of the CD8+ Tcm cells both in blood and lymph node from healthy donor and NSCLC patients (Fig. 1d). In addition, the fraction of the CD8+ Teff cells was higher than that of the CD4+ Teffs in the NSCLC-PBMC (*p* = 0.0003), HD-PBMCs (*p* = 0.0004), Normal-Ly (*p* = 0.0181), and NSCLC-Ly (*p* = 0.003) (Fig. 1d). The percentage of Tem cells did not differ between the CD4 and CD8 groups in HD-PBMC, Normal-Ly, and NSCLC-PBMC, while the CD4+ Tem cells outnumbered the CD8+ Tem cells in NSCLC-Ly (*p* = 0.0049) (Fig. 1d). Our results indicate that in human NSCLC patients, CD4+ and CD8+ T cell subsets show characteristic patterns of distribution that differ among the tissues.

### IFN-γ and TNF-α production is significantly decreased in the naïve CD8 T cells of NSCLC patients

**Fig. 2:**
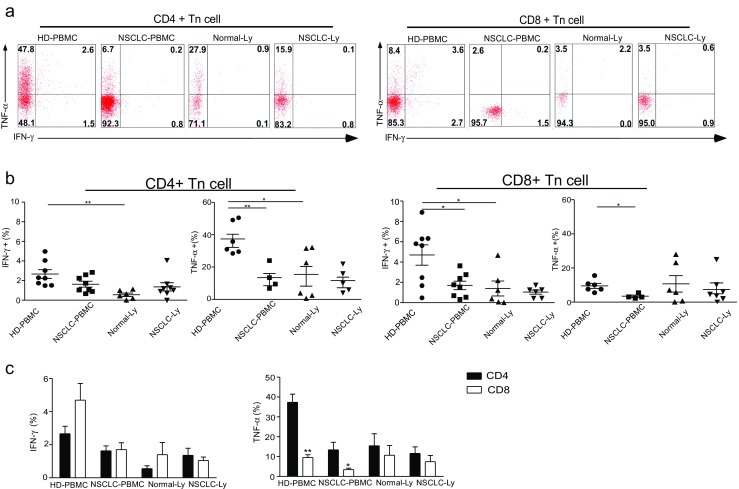
IFN-γ and TNF-α production is significantly decreased in the naïve CD8 T cells of NSCLC patients. PBMCs or lymphocytes isolated from the blood were stimulated for 4-6 hr with PMA + Ionomycin, and cytokine production was assessed by intracellular cytokine staining (ICS). **a** Representative ICS analysis of cytokine production by CD4+CD45RA+CCR7+ and CD4-/CD8+CD45RA+CCR7+ T cells in the blood relative to the unstimulated controls. The numbers in the quadrants indicate the percent of CD4+ T cells or CD8+ T cells that produced IFN-γ, TNF-α, IL-17 or both cytokines. **b** The mean IFN-γ and TNF-α production (±SEM) of the naïve CD4+ T cells (*left*) and CD8+ T cells (*right*) in the blood and lymph node from the non small cell lung cancer patients and healthy donors. *p < 0.05; **p < 0.005; non-paired Student’s t-test. **c** The events of IFN-γ, TNF-α–expressing CD4+ Tn and CD8+ Tn cells in the blood and lymph node from non small cell lung cancer patients and healthy donors, expressed as the mean ± SEM. *p < 0.05; ***p < 0.001; Mann–Whitney test (two-tailed) and non-paired Student’s t-test

The striking differences in the function of naïve and memory T cells occur via the great diversity of cytokines produced within hours of stimulation. We examined whether the functional capacities of the naïve CD4+ or CD8+ T cells differed between NSCLC patients and the healthy controls. After a short-term stimulation with PMA plus ionomycin, the production of IFN-γ, TNF-α, and IL-17 was investigated. Only a small proportion (about 4%) of the naïve CD4+ T cells from the blood of healthy donor produced IFN-γ (Fig. 2a), and no significant difference was observed between healthy donors and the NSCLC patients in IFN-γ production by the CD4+ Tn cells from the blood (Fig. 2b). The proportions of TNF-α-expressing cells were observed in the CD4+ Tn cell population in the blood of healthy donors (Fig. 2a). In contrast, in the peripheral blood of the NSCLC patients, the proportion of TNF-α-expressing cells decreased to below 20%. There were remarkably fewer CD8+ Tn cells that secreted IFN-γ (*p* = 0.0158), TNF-α (*p* = 0.0121), or both in the NSCLC-PBMC than in the HD-PBMC (Fig. 2b). We did not detect IL-17 production from naïve CD4+ or CD8+ T cells in the blood and lymph node of healthy donors and NSCLC patients (Supplementary 1). There was no difference in the IFN-γ and TNF-α production of naïve T cells between NSCLC-Ly and Normal-Ly (Fig. 2b). These results indicate that few of the naïve T cells in the blood secrete IFN-γ. Naïve CD4+ T cells in the blood can secrete abundant TNF-α and were significantly increased compared with naïve CD8+ T cells in healthy donor (*p* = 0.0021), and the levels of TNF-α were significantly decreased in the NSCLC patients (*p* = 0.0402) (Fig. 2c). Negligible IL-17 production from the naïve T cells was found for both sample types from the healthy donors and the NSCLC patients.

### Cytokine-expressing CD4+ Tcm cells are decreased in blood of human NSCLC patients

**Fig. 3:**
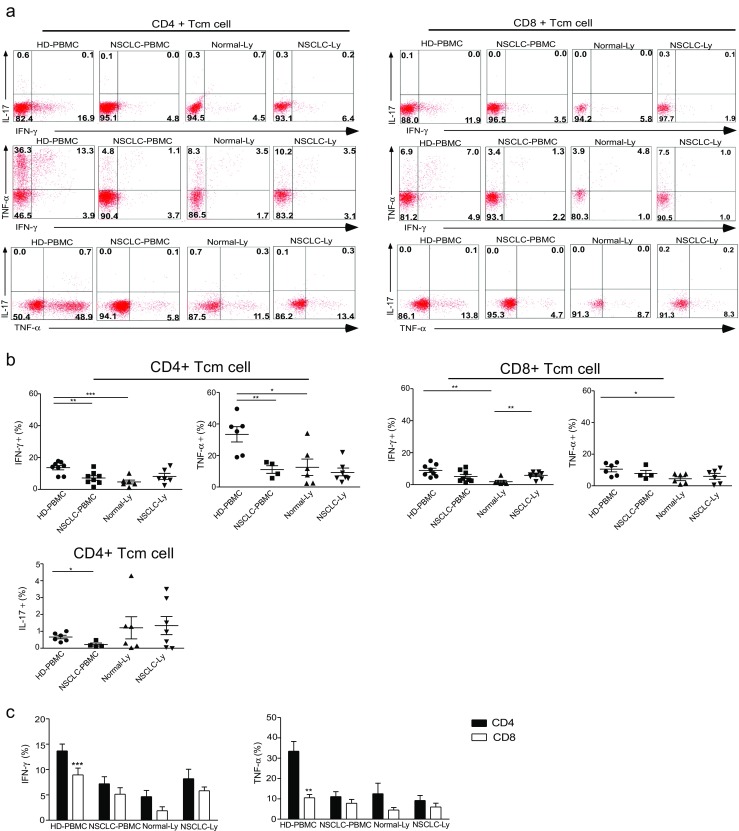
Cytokine-expressing CD4+ Tcm cells are decreased in blood of human NSCLC patients. **a** Flow cytometry plots showing IL-17, IFN-γ, and TNF-α expression in the CD4+CD45RA−/CD45RO+CCR7+ and CD4−/CD8+CD45RA−/CD45RO+CCR7+ T cells from the blood and lymph node of the non-small cell lung cancer patients and healthy donors. **b** The mean frequency (±SEM) of the IFN-γ-expressing, TNF-α-expressing, and IL-17-expressing T cells gated on the CD4+ Tcm and CD4−/CD8+ Tcm cells in the blood. **c** The graph shows the relative frequencies of the cytokine-producing CD4+ Tcm and CD8+ Tcm cells in the HD-PBMC, LC-PBMC, Normal-Ly, and NSCLC-Ly. *p < 0.05, **p < 0.005, ***p < 0.001; non-paired Student’s *t* test

A hallmark of memory T cells is their rapid recall response to stimulation. In humans, the definition of Tcm and Tem cells is based on two distinct criteria: the absence or presence of the immediate effector function and the expression of homing receptors that allow the cells to migrate to secondary lymphoid organs versus non-lymphoid tissues. Human Tcm cells are CD45RO+CCR7+ cells and produce large amounts of IFN-γ, IL-2, and IL-4 [2]. We examined the production of IFN-γ, TNF-α, and IL-17 by the CD4+ or CD8+ Tcm cells in the blood of the NSCLC patients after 4 h of stimulation with PMA plus ionomycin (Fig. 3a). Higher frequencies of IFN-γ, TNF-α, and/or dual-expressing CD4+ Tcm cells were observed in the blood from the healthy donors, and similar proportions of cells that produced these cytokines were observed in the CD8+ Tcm population (Fig. 3a). Compared to the NSCLC patients, the frequency of IFN-γ, TNF-α, and/or double-positive CD4+ Tcm cells in the blood was higher in the healthy donors (*p* < 0.01) (Fig. 3b). IL-17 production after PMA plus ionomycin stimulation was observed, albeit at a low frequency; the fraction of IL-17-secreting CD4+ Tcm cells was lower in NSCLC-PBMC than in HD-PBMC (*p* = 0.0166) (Fig. 3b). We did not detect IL-17 expression in the CD8+ Tcm cells (Supplementary 2). The frequency of the IFN-γ-expressing and TNF-α-expressing CD4+ Tcm cells was greater than that of IFN-γ-expressing (*p* = 0.0007) and TNF-α-expressing CD8+ Tcm cells (*p* = 0.002) in the HD-PBMC (Fig. 3c). Furthermore, there was a higher proportion of IFN-γ-expressing CD8+ Tcm cells in the NSCLC-Ly than in the Normal-Ly (*p* = 0.0038), and there was a higher proportion of the IFN-γ-expressing and TNF-α-expressing Tcm cells in the HD-PBMC (*p* = 0.0013) than in Normal-Ly (*p* = 0.016) (Fig. 3b). Together, these results show that the IFN-γ-expressing, TNF-α-expressing, and IL-17-expressing CD4+ Tcm cells were significantly decreased in the blood of the NSCLC patients.

### IFN-γ-expressing CD8+ Teff cells are remarkably reduced in the blood of NSCLC patients

**Fig. 4:**
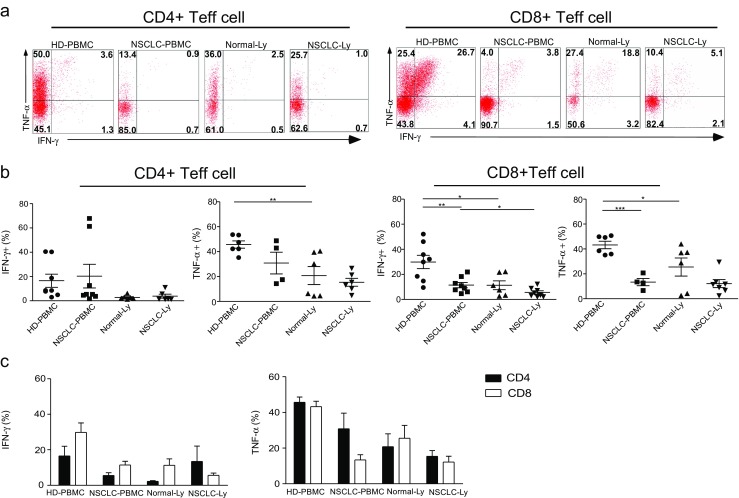
IFN-γ-expressing CD8+ Teff cells are remarkably reduced in blood of NSCLC patients. **a** IFN-γ and TNF-α dual expression by CD4+CD45RA+CCR7− (*left*) and CD4−/CD8+CD45RA+CCR7− (*right*) effector T cells in the blood and lymph node from non-small cell lung cancer patients and healthy donors. **b** The mean IFN-γ and TNF-α production (±SEM) in the cells from the blood of between healthy donor and NSCLC patients. *p < 0.05, **p < 0.005, ***p < 0.001; non-paired Student’s *t* test. **c** The percentage of IFN-γ-producing and TNF-α-producing CD4+ Teff and CD8+ Teff cells in the blood and lymph node from non-small cell lung cancer patients and healthy donors, expressed as the mean ± SEM

Effector cells secrete higher titers of cytokines than either naïve or memory cells and with faster kinetics than the resting T cell populations. IL-17 production in PMA plus ionomycin-stimulated Teff cells was not observed in PBMCs and lymph node from the healthy donors or the NSCLC patients (Supplementary 3). IFN-γ and TNF-α producers in the blood from the NSCLC patients represented approximately 10% of the CD8+ Teff cells, which was significantly lower compared to the levels observed in the blood from the healthy donors (*p* = 0.0061) (*p* = 0.0001) (Fig. 4a, b). Whereas no difference was observed in the proportion of IFN-γ-expressing and TNF-α-expressing CD4+ Teff between NSCLC-PBMC and HD-PBMC, TNF-α produced by the Teff cells significantly reduced in Normal-Ly than in HD-PBMC (CD4 group, *p* = 0.0096; CD8 group, *p* = 0.049); the frequency of IFN-γ-expressing CD8+ Teff cells was also lower in NSCLC-Ly than in NSCLC-PBMC (*p* = 0.035) (Fig. 4b). There was no difference in the cytokine production between CD4+ Teff and CD8+ Teff in the lymph node and blood (Fig. 4c). Our results therefore demonstrated different patterns of functional CD8+ Teff cells in these tissue sites; in particular, the production of TNF-α and IFN-γ by the CD8+ Teff cells in PBMCs from the NSCLC patients was significantly lower than that of the healthy donors.

### The levels of IFN-γ-expressing, TNF-α-expressing, and IL-17-expressing CD4+ Tem cells are decreased in the blood of NSCLC patients

**Fig. 5:**
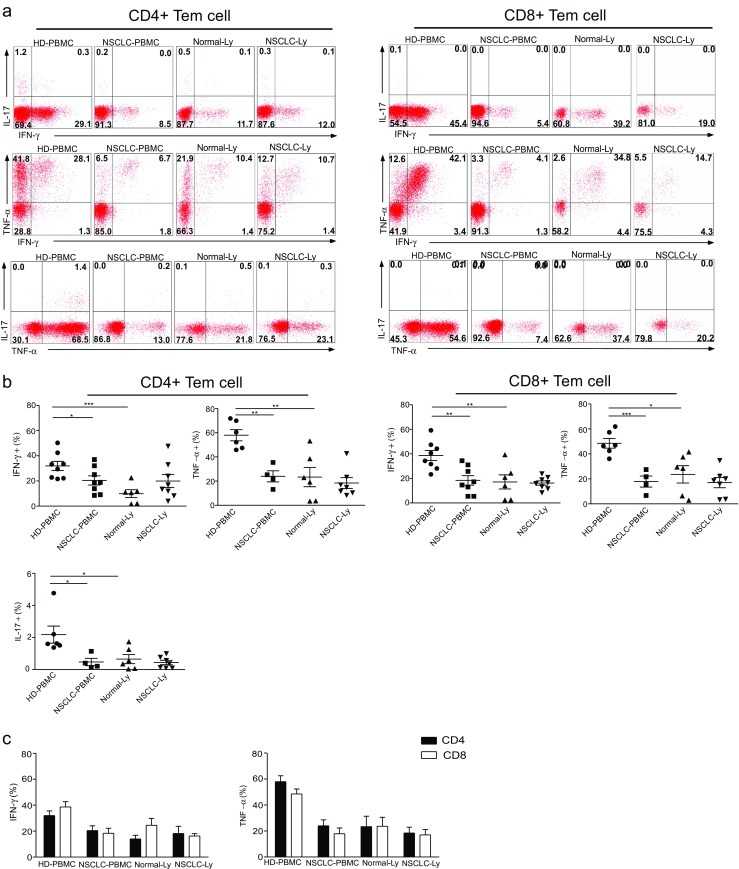
The levels of IFN-γ-expressing, TNF-α-expressing, and IL-17-expressing CD4+ Tem cells are decreased in the blood of NSCLC patients. **a** IFN-γ, TNF-α, and IL-17 production by the CD4+ and CD8+ Tem cells in the blood and lymph node, gated on the unstimulated controls. The *numbers in the quadrants* indicate the percent of the CD4+ T cells (*left*) or CD8+ T cells (*right*) that produce IFN-γ, TNF-α, IL-17, or both cytokines. **b** The mean frequency (±SEM) of the IFN-γ-expressing, TNF-α-expressing, and IL-17-expressing T cells gated on the CD4+ Tem (*left*) and CD8+ Tem (*right*) T cells in the blood. *p < 0.05, **p < 0.005, ***p < 0.001; Mann–Whitney test (two-tailed) and non-paired Student’s *t* test. **c** The plot of IFN-γ-secreting and TNF-α-secreting CD4+ Tem and CD8+ Tem cells in the blood and lymph node from non-small cell lung cancer patients and healthy donors, expressed as the mean ± SEM

Compared with Tcm cells, Tem cells are characterized by a rapid effector function. CD8+ Tem express large amounts of perforin, and both CD4+ and CD8+ produce IFN-γ, IL-4, and IL-5 within hours of antigenic stimulation. IL-17 is primarily produced by the CD4+ Tem cells in the blood (Fig. 5a). IFN-γ, TNF-α, IL-17, and/or dual expressors among the CD4+ and CD8+ Tem cells were found to exceed those of the CD4+ and CD8+ Teff cells in the blood (Fig. 5a). IFN-γ and TNF-α producers of Tem cells were decreased in Normal-Ly than in HD-PBMC (CD4 group, *p* ≤ 0.004; CD8 group, *p* ≤ 0.01) (Fig. 5b). The levels of IFN-γ, TNF-α, and IL-17 cytokine-expressing CD4+ and CD8+ Tem cells were decreased in the blood from the NSCLC patients compared to the healthy donors (CD4 group, *p* ≤ 0.04; CD8 group, *p* ≤ 0.003) (Fig. 5b, Supplementary 4). In the blood and lymph node of the NSCLC patients, the proportion of cytokine-expressing CD8+ Tem cells and CD4+ T cells showed the same pattern (Fig. 5c). These results demonstrate that human Tem cells are the primary source of IFN-γ, IL-17, and TNF-α cytokine production. In the NSCLC patients, all of the examined cytokines produced by the Tem cells in the blood were significantly decreased.

## Discussion

In this study, we analyzed the distribution and functional capacity of the CD4+ and CD8+ T cell subsets in the blood and lymph node of human NSCLC patients. The frequencies of the CD8+ T cell subsets were similar for the blood and lymph nodes of the NSCLC patients. The CD8+ Teff cells predominated in both tissue sites, followed by the Tem, Tn, and Tcm cells. However, the composition of the CD4+ T cell subsets differed. The most one cell type was CD4+ Tn, followed by Tem, Tcm, and Teff in the NSCLC-PBMC. The CD4+ Tem cells were the major population in the NSCLC-Ly, followed by the Tcm, Teff, and Tn cells. In the functional analysis, we found that the levels of the IFN-γ-expressing CD8+ Tcm cells were increased in the lymph nodes of the human NSCLC patients. The capacity to express IFN-γ was remarkably reduced in the lymph nodes relative to the blood of the NSCLC patients, even though the CD8+ Teff cells were present at a higher frequency in the NSCLC patients than in the healthy donors. The levels of IFN-γ-expressing, TNF-α-expressing, and IL-17-expressing CD4+ Tem and CD4+ Tcm cells were significantly decreased in the blood of the NSCLC patients compared to healthy donors. We observed that all three of the examined cytokines secreted by the CD4+ and CD8+ T cell subsets were present at lower frequencies in the lymph node than in the blood of the healthy donors. Our results showed differences in the composition and function of the CD4+ and CD8+ T cell subsets in the blood and lymph node of NSCLC patients. The identification of these differences may improve our understanding of the role of the T cell-mediated immune response in antitumor immunity.

Different tissue locations and various types of human cancer possess distinct distributions of T cell subsets. Kuss showed that there was an increase in the effector CD8+ T (CD8+CD27−CD45RA−) population in the peripheral blood from head and neck carcinoma patients [15]. Another group observed that there were no significant differences between the effector CD8+ T cells (CD8+CD27−CD45RA−) in the peripheral blood of healthy donors and lung adenocarcinoma patients [16]. However, there was an elevated population of memory (CD45RA−CD45RO+CD27+CD28+) CD8+ T cells and a low proportion of terminally differentiated (CD45RA+CD45RO−CD27−CD28−) CD8+ T cells in the pleural effusions. These results are similar to the data from the TILs of NSCLC patients in whom the CD4+ T cell subpopulation is increased [10]*.* In our analysis of the CD8+ and CD4+ T cell subsets in the blood and lymph node from NSCLC patients, we identified the Tn, Tcm, Tem, and Teff cells according to established surface markers [1, 6] in eight NSCLC patients. We found that the levels of the Teff CD8+ T cells were significantly elevated in the blood from the NSCLC patients, and these cells were also present in a higher frequency in the lymph node. CD8+ T cells play an important role in the cell-mediated antitumor immune response [17]. However, the role of the CD8+ Teff cells in the lymph node is unclear. The CD4+ T cell response is essential in preventing the induction of tolerance by tumor antigens, and it helps the CD8+ T cells differentiate into sustainable memory cells [12] that can initiate antitumor immune responses. Importantly, the numbers of CD4+ T cells are positively correlated with a favorable prognosis in lung cancer patients [18]. Our results indicate that the subtypes of the CD4+ and CD8+ Tm cells in NSCLC patients are distinct, and a lower proportion of CD8+ Tcm cells, compared to CD4+ Tcm cells, was found in both the peripheral blood and the lymph node from NSCLC patients. In contrast, a lower frequency of the CD8+ Tem cells, compared to the CD4+ Tem cells, was observed only in the lymph node. The mechanism by which CD4+ T cells aid in the formation of CD8+ memory T cells remains unclear. CD4+ effector T cells can mediate direct tumor destruction alone or with the help from CD8+ T cells [9, 18–20]. However, the role of the CD4+ memory T cell subsets in the antitumor response needs to be further clarified. In humans, Tcm cells migrate to lymphoid tissue, while Tem cells circulate to the non-lymphoid tissues [2, 21, 22]. We found that in the lymph node, the population of the Tem cells was higher than that of the Tcm cells. The high proportion of the Tem cells in the lymph node might be due to a replenishment of the high frequency of recycling Teff cells.

TNF-α-expressing and IFN-γ-expressing naïve, memory, and effector T cells were observed with low frequencies in the blood of the NSCLC patients. IL-17 production was only observed in the CD4+ Tcm and Tem cells of NSCLC subjects.

IFN-γ is the hallmark cytokine of Th1 cells and CD8+ T cells, and it is critical for immune surveillance [23]. The function of the CD8+ T cells from lung cancer patients was impaired with respect to both Th1 cytokine production and cytotoxic potential [23]. However, our results showed that the proportion of the IFN-γ-producing CD8+ Tcm cells was increased in the lymph node from the NSCLC patients. IFN-γ production by the CD8+ Teff cells was significantly decreased in the blood from the NSCLC patients, yet the number of CD8+ Teff cells was increased. Tcm cells can differentiate into Tem and Teff cells. It is possible that the CD8+ Tcm cells replenish the Teff cells, leading to the high frequency of circulating CD8+ Teff cells observed in the blood of the lung cancer patients. In addition to IFN-γ, CD8+ Teff cells also express the perforin, granzyme to kill tumor cells. Studies have found that the IFN-γ-producing Th1 and CD8+ T cells are more prone to apoptosis and are involved in the reduction of the Teff cell populations [24, 25]. The IFN-γ-deficient CD8+ T cells that expressed high levels of IL-7r were shown to be the precursors of the memory cells. These cells block the IFN-γ signaling pathway that contributes to the memory responses involved in tumor vaccination [26–28]. The role of the IFN-γ-producing CD8+ Teff cells during the formation of the CD8+ T memory cells in human lung cancer is less clear.

A role for inflammation in tumorigenesis is now generally accepted, and it has become evident that an inflammatory microenvironment is an essential component of all tumors [29]. The cytokines in the tumor microenvironment can either promote antitumor immunity (IL-12, IFN-γ), enhance tumor development and progression (IL-6, IL-17, IL-23) [30], or influence the cancer cell growth and survival (TRAIL, FasL, TNF-α, TGF-β, IL-6). TNF-α in the bloodstream may have oncogenic effects through several pathways, such as the stimulation of the production of reactive oxygen species (ROS), which can induce DNA damage and genomic instability; the stimulation of stem cell-like tumor progenitors by promoting β-catenin entry into the nucleus in inflammation-associated gastric cancer [31]; and the promotion of MMP expression, the invasiveness, and the survival of circulating metastatic seeds via NF-κB and STAT3 [32, 33]. We found that the frequency of TNF-α production in the Tcm, Tem, Tn, and Teff cells from the blood of lung cancer patients is lower than that of healthy donors. The low circulating levels of TNF-α in the bloodstream might have beneficial effects in lung cancer patients. Whether these low levels are one of the protective mechanisms in human lung cancer needs to be verified.

The role of IL-17 in antitumor processes remains controversial. Some studies have reported that the proportion of Th17-producing cells was higher in multiple human cancers and that these cells have a potent antitumor effect. This effect might be related to the polyfunctional effector cytokines induced by the IL-17 cells, specifically the induction of TNF-α, IL-2, IFN-γ, and chemokines and the recruitment of NK cells into the tumor microenvironment to target the tumor [34–36]. Other reports have shown that IL-17 induces tumor angiogenesis [37, 38] and that a high level of IL-17 is correlated with advanced cancer [39]. Our current data revealed that IL-17 was produced primarily by the CD4+ Tcm and CD4+ Tem cells, not by other CD4+ and CD8+ T cell subsets, which is consistent with the reports that 99% of the tumor-infiltrating IL-17 T cells were IL-17 CD4+ (Th17) cells [40, 41]. In addition, the proportion of the IL-17 CD4+ memory T cells was decreased in the blood from the NSCLC patients relative to the healthy donors. The number of cells positive for dual cytokines, including IL-17/IFN-γ and IL-17/TNF-α in the blood of the NSCLC patients, was lower than in the healthy donors. The effect of IL-17 production on the CD4+ memory T cells in human lung cancer requires further investigation.

## Electronic supplementary material

ESM 1 (PDF 32 kb)

ESM 2 (PDF 30 kb)

ESM 3 (PDF 32 kb)

ESM 4 (PDF 30 kb)
