# Supplementary figures and images for: The distribution and function of human memory T cell subsets in lung cancer
Source: Immunol Res. 2017 Jan 19;65(3):639–50. doi: 10.1007/s12026-016-8882-y (PMC5440487; doi:10.1007/s12026-016-8882-y)

## Supplementary 1

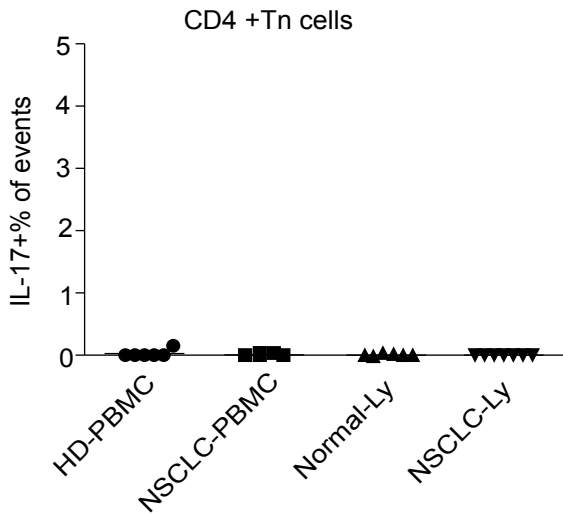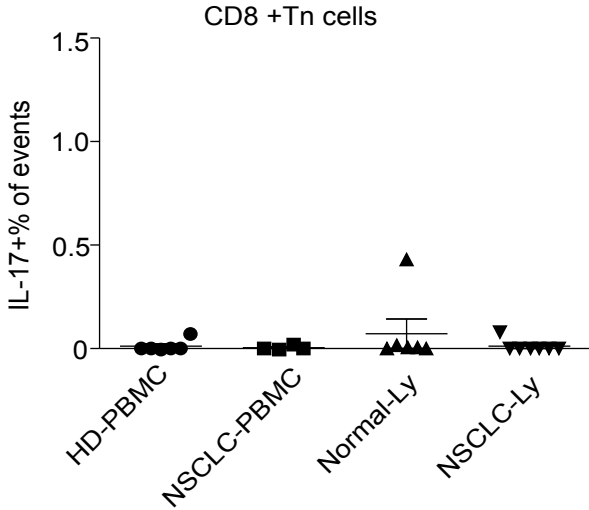

Supplement: Supplementary file 1 — (PDF 32 kb) [file 12026_2016_8882_MOESM1_ESM.pdf]

Supplementary 2

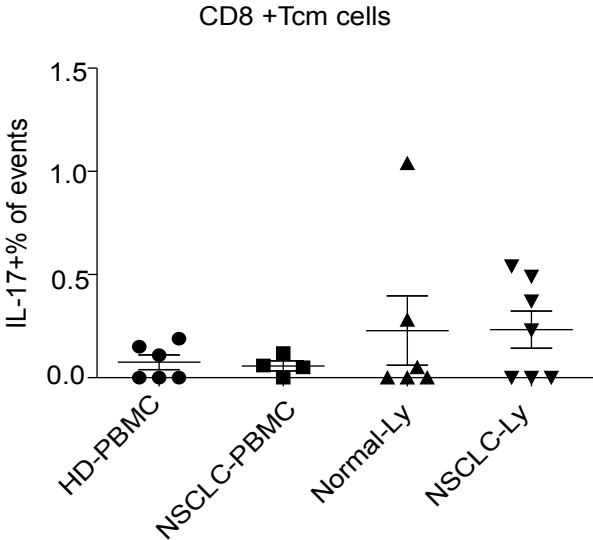

Supplement: Supplementary file 2 — (PDF 30 kb) [file 12026_2016_8882_MOESM2_ESM.pdf]

Supplementary 3

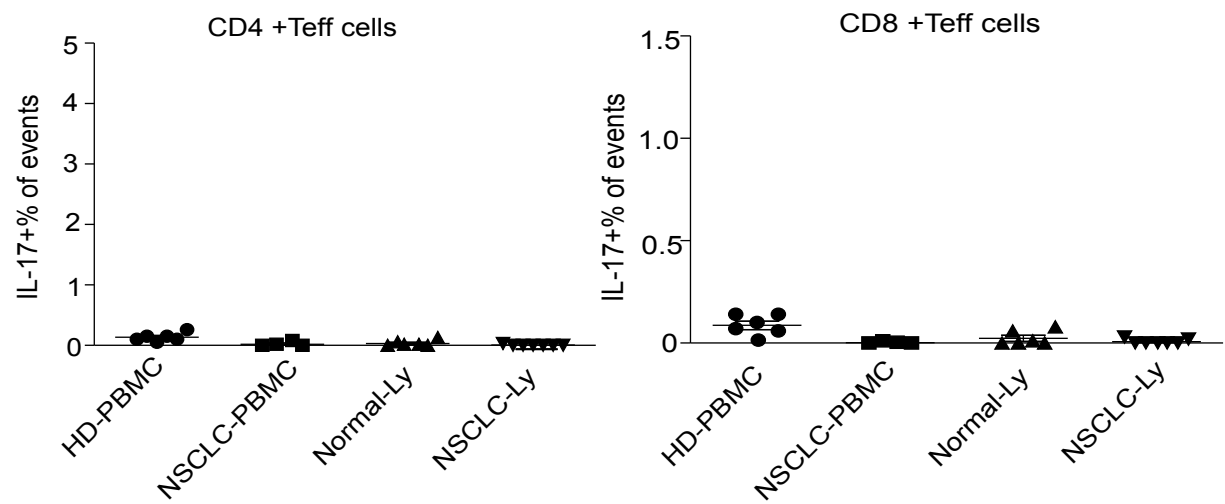

Supplement: Supplementary file 3 — (PDF 32 kb) [file 12026_2016_8882_MOESM3_ESM.pdf]

Supplementary 4

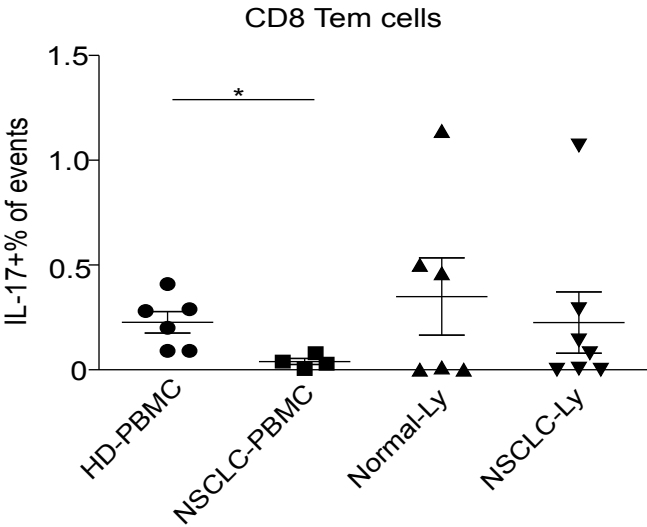

Supplement: Supplementary file 4 — (PDF 30 kb) [file 12026_2016_8882_MOESM4_ESM.pdf]
